# Supplementary material for: HPLC Study of Product Formed in the Reaction of NBD-Derived Fluorescent Probe with Hydrogen Sulfide, Cysteine, N-acetylcysteine, and Glutathione
Source: Molecules. 2022 Nov 28;27(23):8305. doi: 10.3390/molecules27238305 (PMC9736530; doi:10.3390/molecules27238305)
Supplement: Supplementary file 1 [file molecules-27-08305-s001.zip › molecules-2045040-supplementary.pdf]

Supplementary Materials

HPLC Study of Product Formed in the Reaction of NBD-Derived Fluorescent Probe with Hydrogen Sulfide, Cysteine, *N*-acetylcysteine, and Glutathione

Daniel Słowiński [1], Małgorzata Świerczyńska [1], Jarosław Romański [2] and Radosław Podsiadły [1,*]

[1] Institute of Polymer and Dye Technology, Faculty of Chemistry, Lodz University of Technology, Stefanowskiego 16, 90-537, Lodz, Poland

[2] Department of Organic and Applied Chemistry, Faculty of Chemistry, University of Lodz, Tamka 12, 91-403, Lodz, Poland

[*] Correspondence: radoslaw.podsiadly@p.lodz.pl

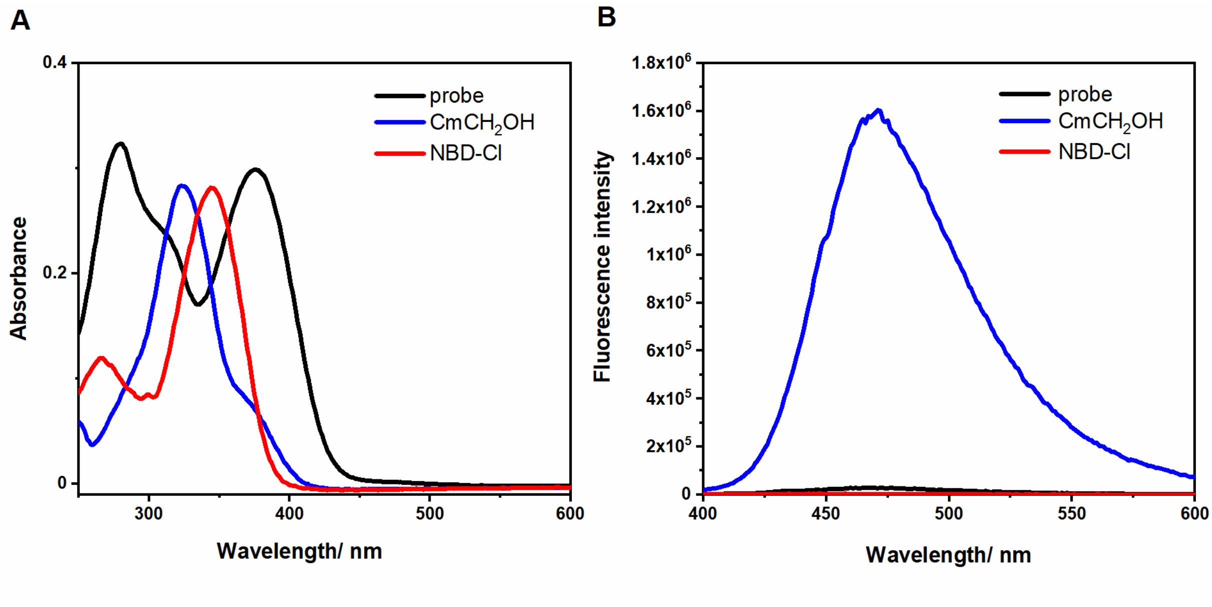

Figure S1. (A) UV-Vis absorption and **(B)** emission spectra of the **NBD-O-CmCH$_2$OH** probe, **CmCH$_2$OH,** and **NBD-Cl** recorded in phosphate buffer (0.1 M, pH 7.4) containing MeCN (10%). λ_{ex}=320 nm, slites 1.0/1.0 nm.

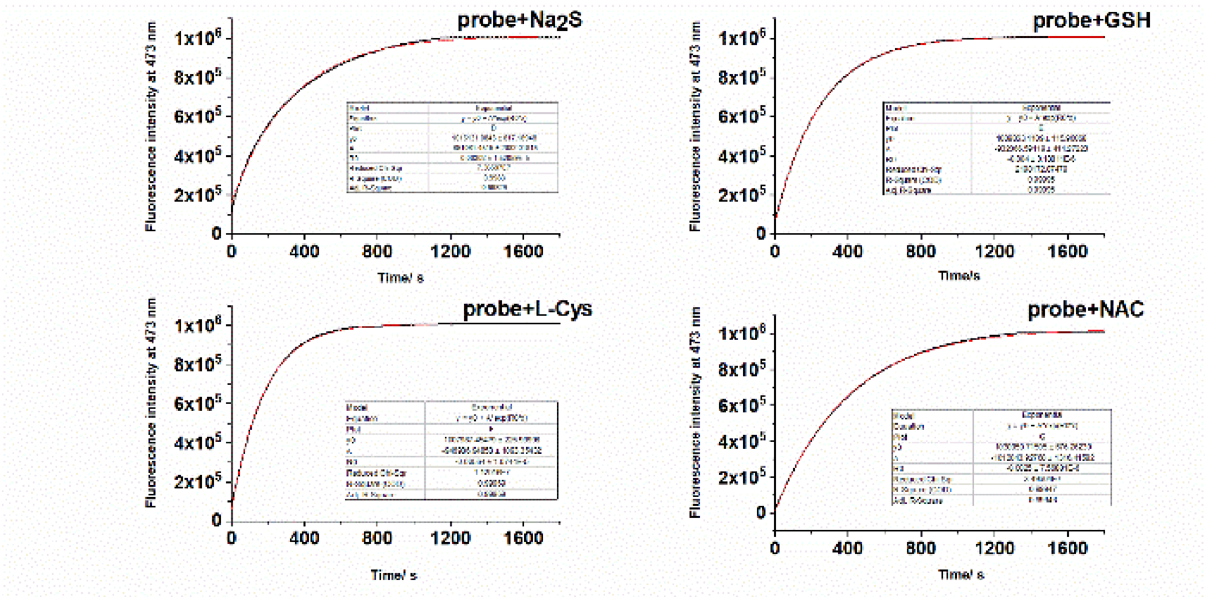

Figure S2. Time-dependent fluorescence intensity changes of **NBD-O-CmCH$_2$OH** (5 μM) at 473 nm when treated with various thiol species (50 μM) in PB buffer.

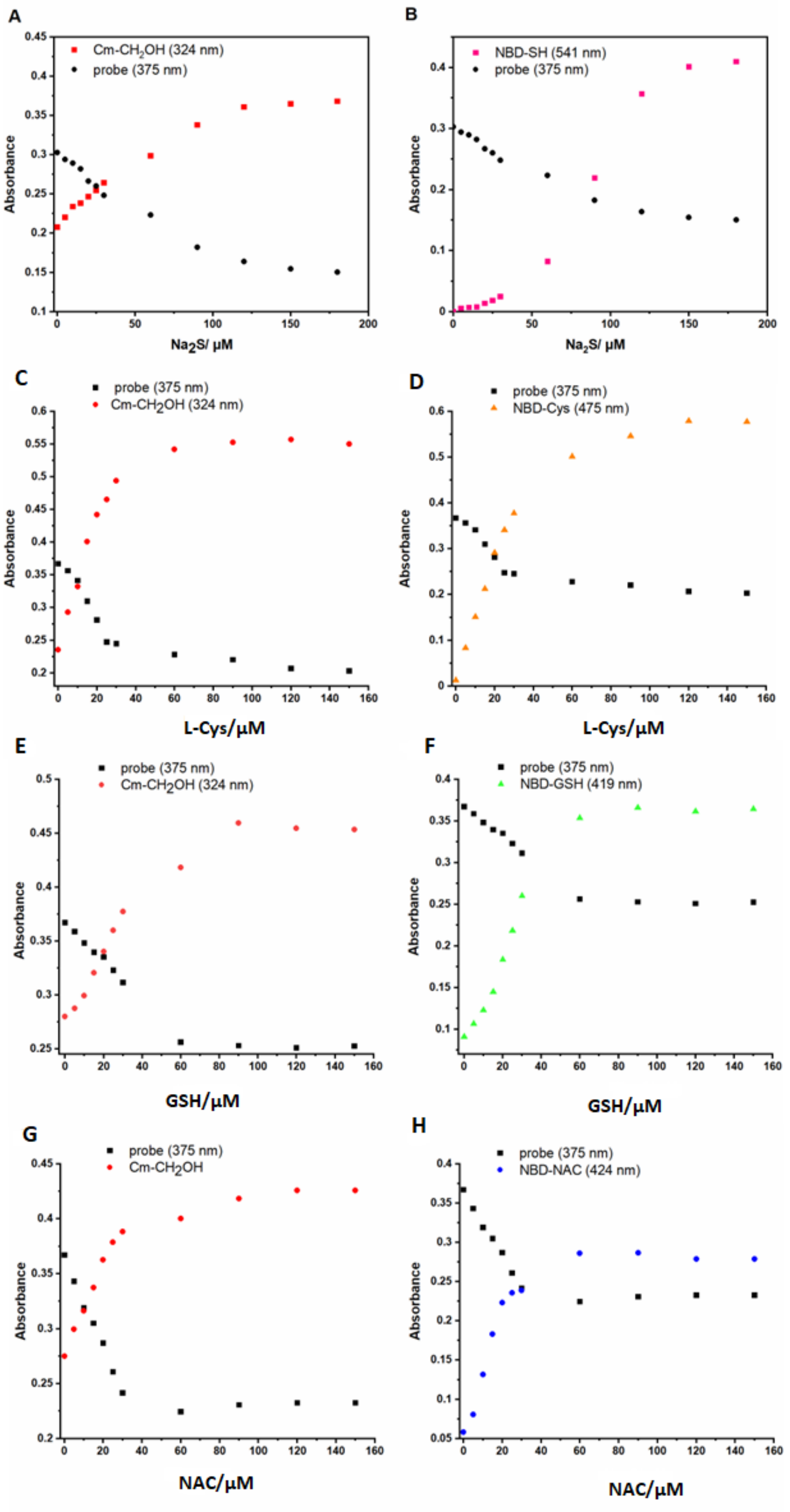

Figure S3. Changes in the characteristic absorption bands during the reaction between the **NBD-O-Cm CH_2OH** probe (30 µM) and (A,B) Na_2S, (C,D) L-Cys, (E,F) GSH (G,H) NAC.

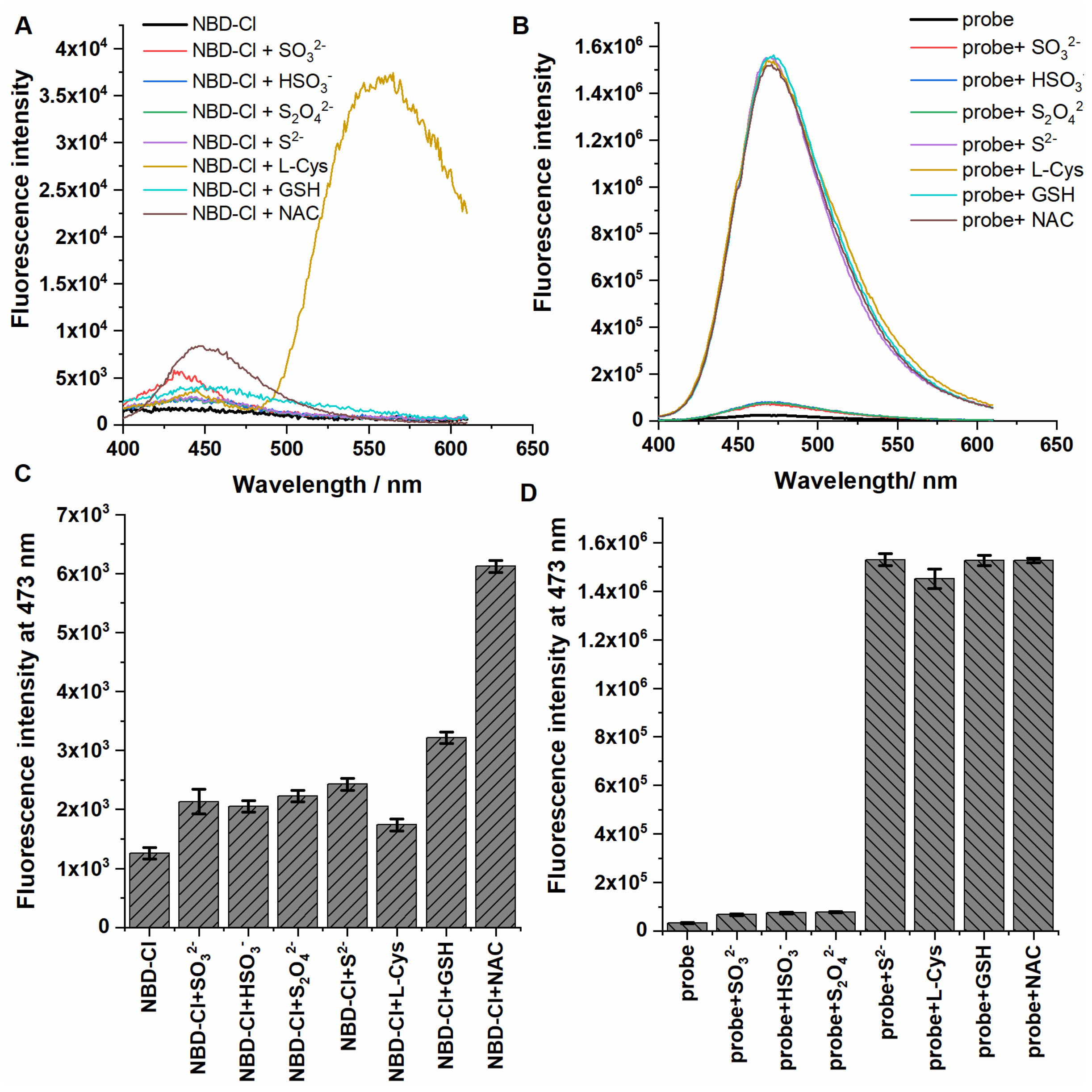

Figure S4. The fluorescence intensity changes of (**A,C**) NBD-Cl and (**B,D**) probe **NBD-O-CmCH$_2$OH** (7.5 µM) upon the addition of sulfur species (37.5 µM for each) in MeCN-PB (1:9. v/v, pH 7.4).

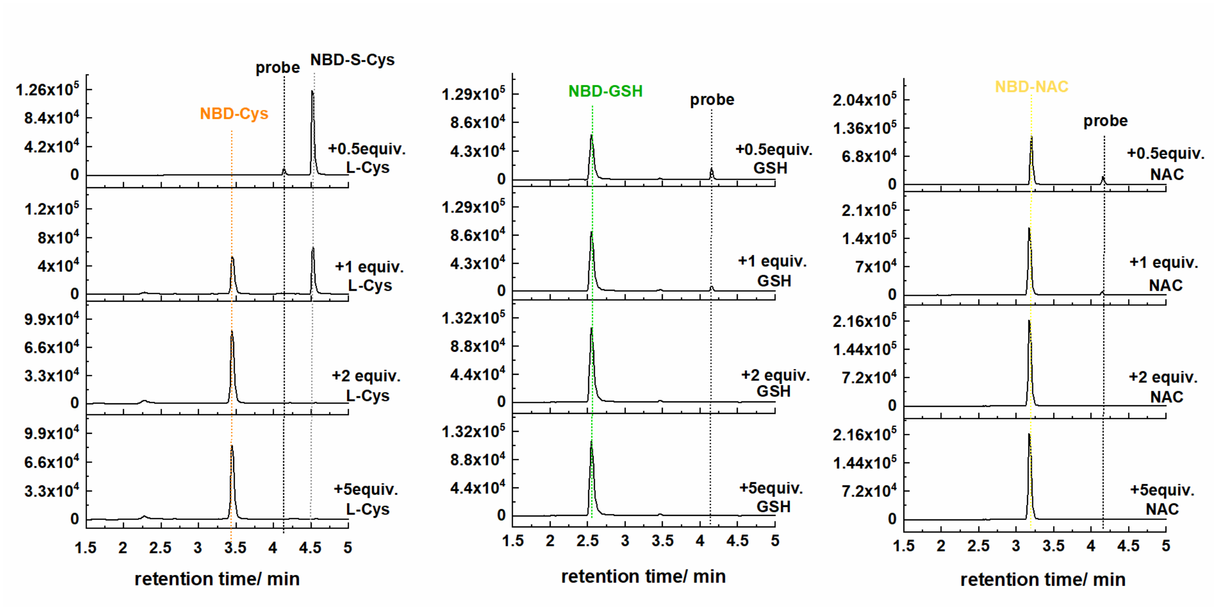

Figure S5. HPLC chromatogram of the reaction mixtures of **NBD-O-CmCH$_2$OH** (60 µM) with **(A)** L-Cys (30 – 300 µM), **(B)** GSH 30– 300 µM, **(C)** NAC 30 – 300 µM after 15 min incubation. The traces were collected using an absorption detector set at 420 nm.

Table 1. Comparison of **NBD-O-CmCH$_2$OH** with the fluorescent probes previously reported.

Structures	Medium	Detection targets	λ_{em} (nm)	λ_{ex} (nm)	Response time (min)	LOD (µM)	Ref.
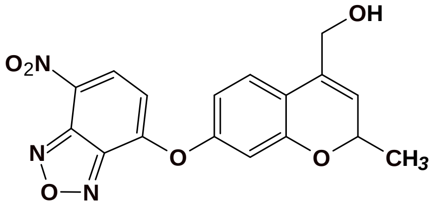 **NBD-O-CmCH$_2$OH**	MeCN:PB (1:9, v/v)	Cys<br>GSH<br>H_2S<br>NAC	320	473	15	0.14<br>0.06<br>0.03<br>0.03	Our work
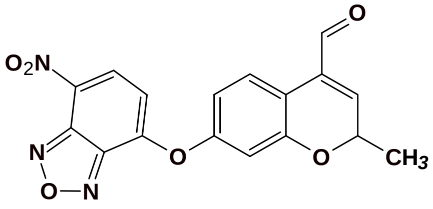 **FHC-O-NBD**	MeCN:PBS (6:4, v/v)	Cys<br>GSH<br>H_2S<br>NAC	340	486	20<br>120<br>ND*<br>ND*	0.11<br>0.79<br>0.42<br>ND*	[S1]

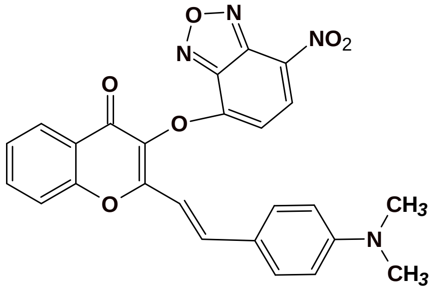<br>**NBD-OF**	MeCN:PBS<br>(3:7, v/v)	Cys<br>GSH<br>H_2S<br>NAC	488	545,621<br>621<br>ND*<br>ND*	60<br>120<br>ND*<br>ND*	2.1<br>6.4<br>ND*<br>ND*	[S2]
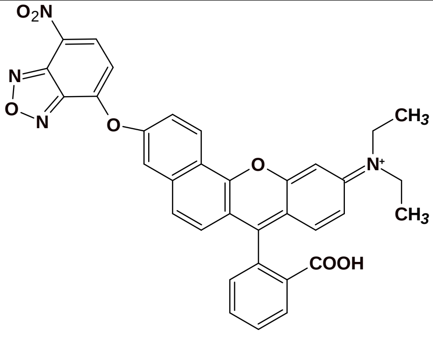<br>**SNARF-NBD**	MeCN:PBS<br>(3:7, v/v)	Cys<br>GSH<br>H_2S<br>NAC	435	543<br>543<br>624<br>ND*	10<br>15<br>ND*<br>ND*	0.05<br>0.06<br>0.06<br>ND*	[S3]
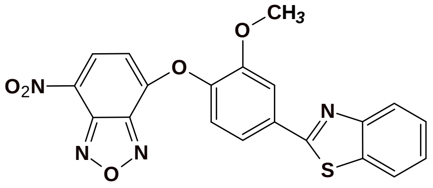<br>**BMNO**	MeCN:HEPES<br>(1:1, v/v)	Cys<br>GSH<br>H_2S<br>NAC	330	405	ND*	1.8<br>1.6<br>ND*<br>ND*	[S4]
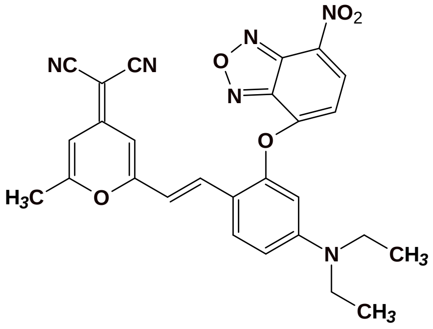<br>**RED-NBD**	MeCN:PB<br>(3:7, v/v)	Cys<br>GSH<br>H_2S<br>NAC	450	560, 630<br>630<br>ND*<br>ND*	10<br>10<br>ND*<br>ND*	0.02<br>0.03<br>ND*<br>ND*	[S5]
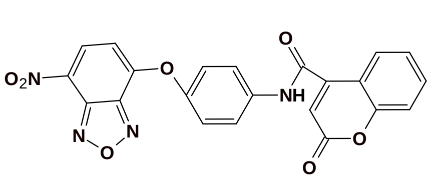<br>**NC-NBD**	DMF:PBS<br>(1:9, v/v)	Cys<br>GSH<br>H_2S<br>NAC	420	520	2<br>ND*<br>ND*<br>ND*	0.43<br>0.36<br>ND*<br>ND*	[S6]

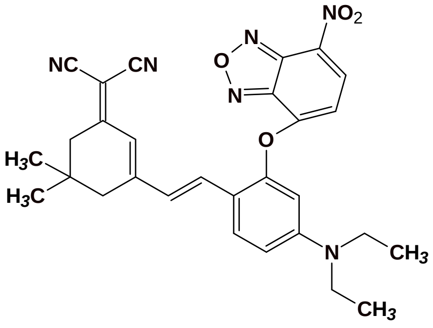<br>**NIR-NBD**	DMF:PBS<br>(4:6, v/v)	Cys<br>GSH<br>H_2S<br>NAC	588	756	15<br>8<br>ND*<br>ND*	0.16<br>0.56<br>ND*<br>ND*	[S7]
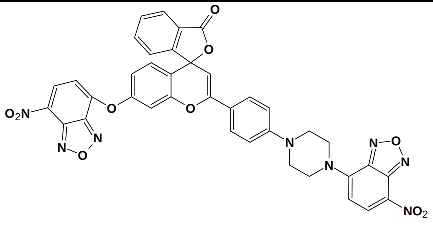<br>**NJB**	MeCN:PBS<br>(1:1, v/v)	Cys<br>GSH<br>H_2S<br>NAC	478<br>ND*<br>546<br>ND*	553<br>ND*<br>604<br>ND*	30<br>ND*<br>180<br>ND*	0.06<br>ND*<br>0.08<br>ND*	[S8]

*ND-no determined

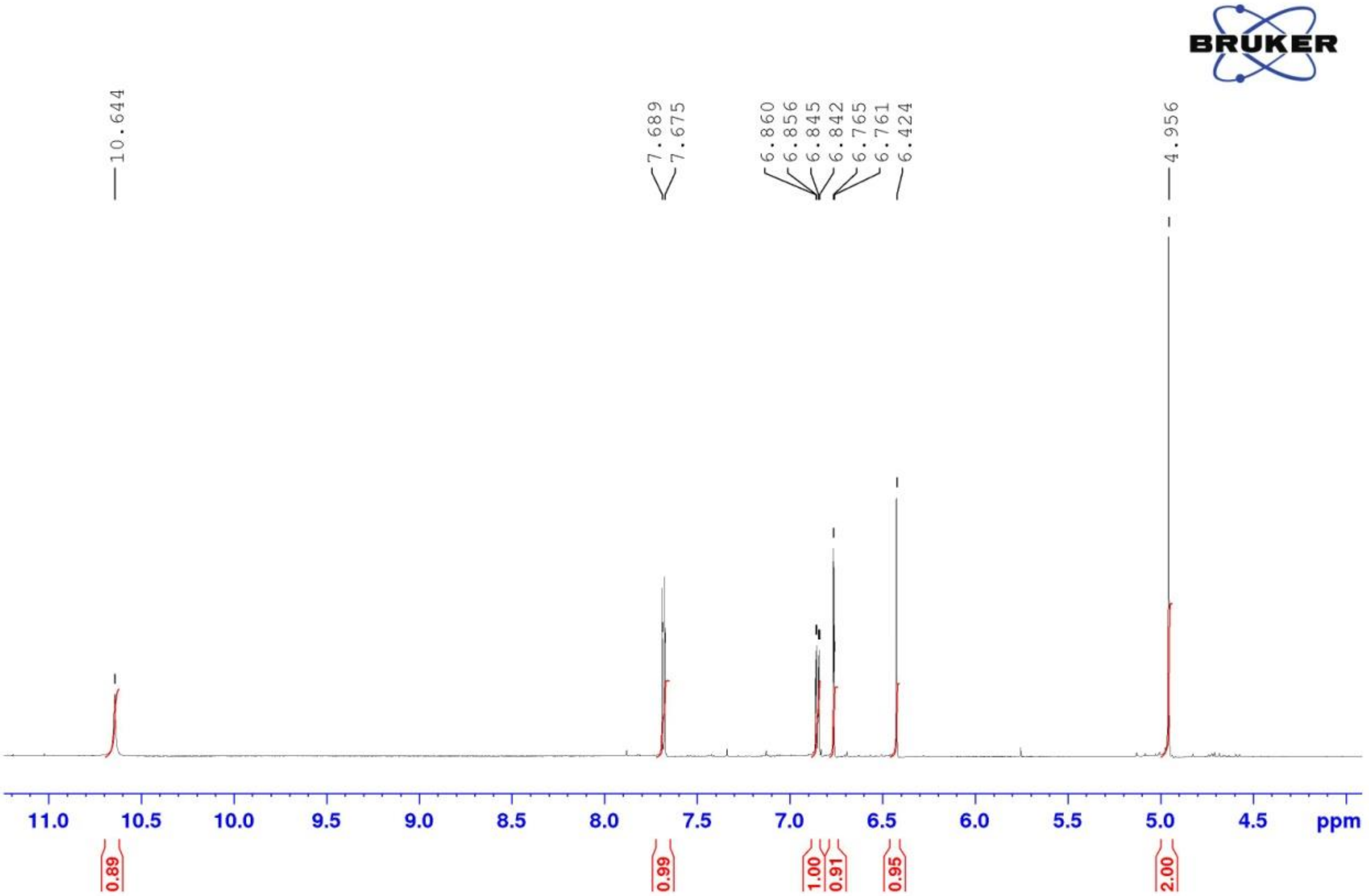

Figure S6. ^{1}H NMR spectrum of **$CmCH_2Cl$**

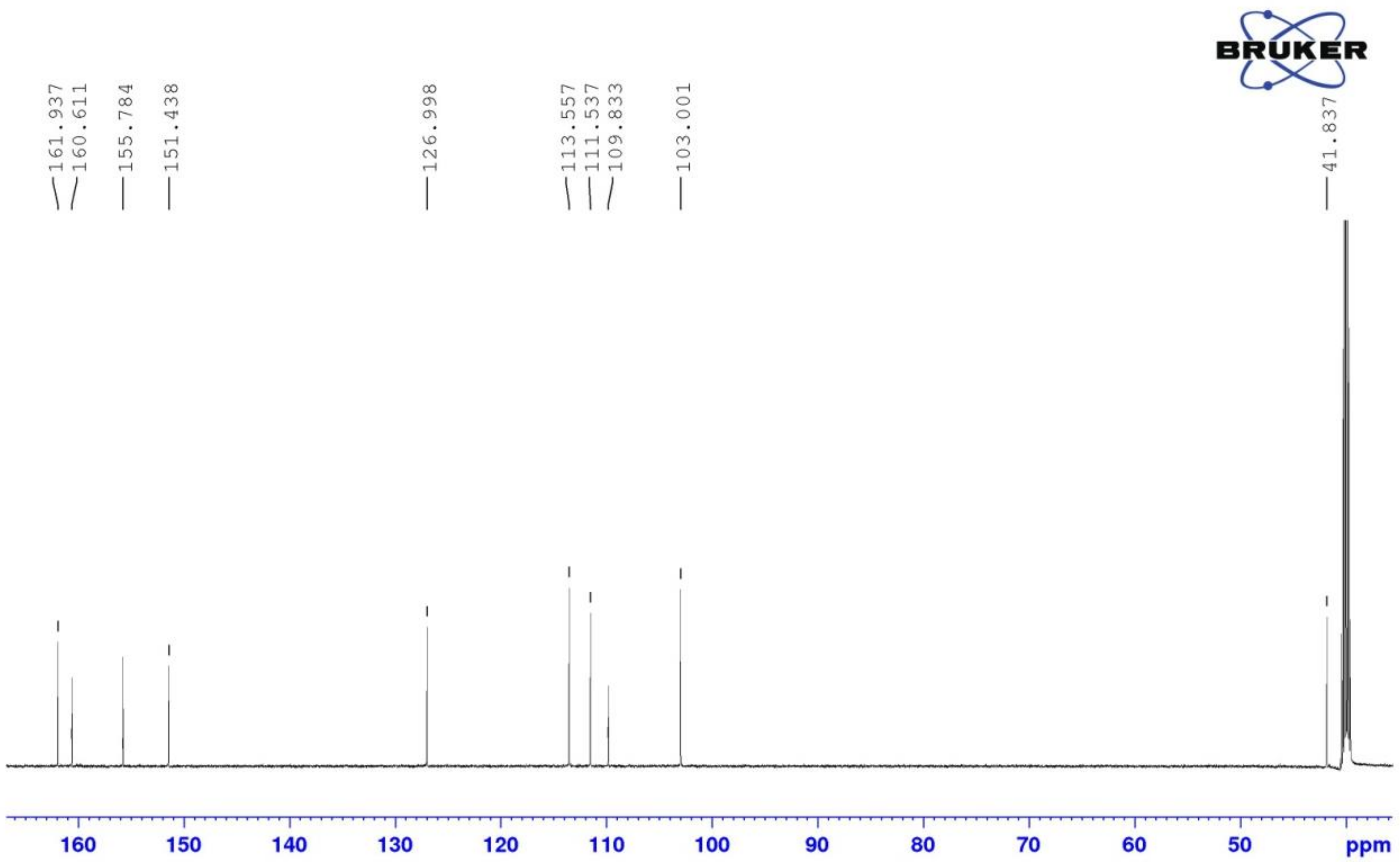

Figure S7. ^{13}C NMR spectrum of **CmCH$_2$Cl**

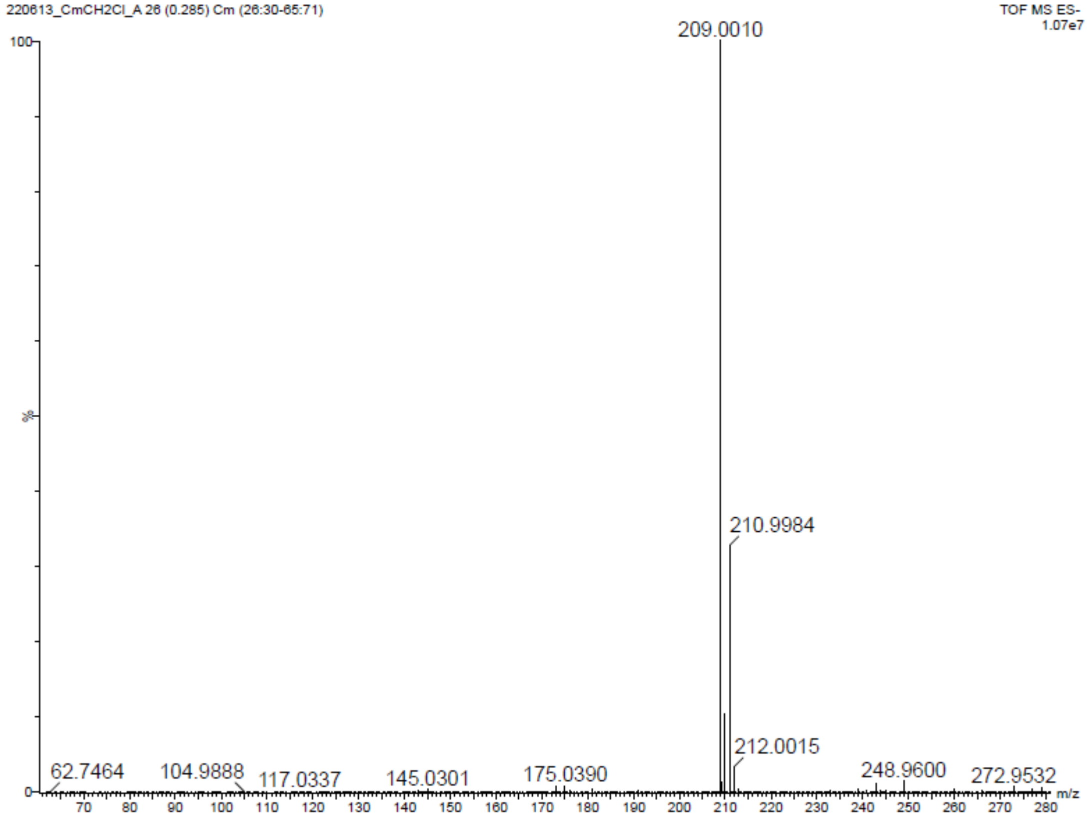

Figure S8. HRMS spectrum of **$CmCH_2Cl$**

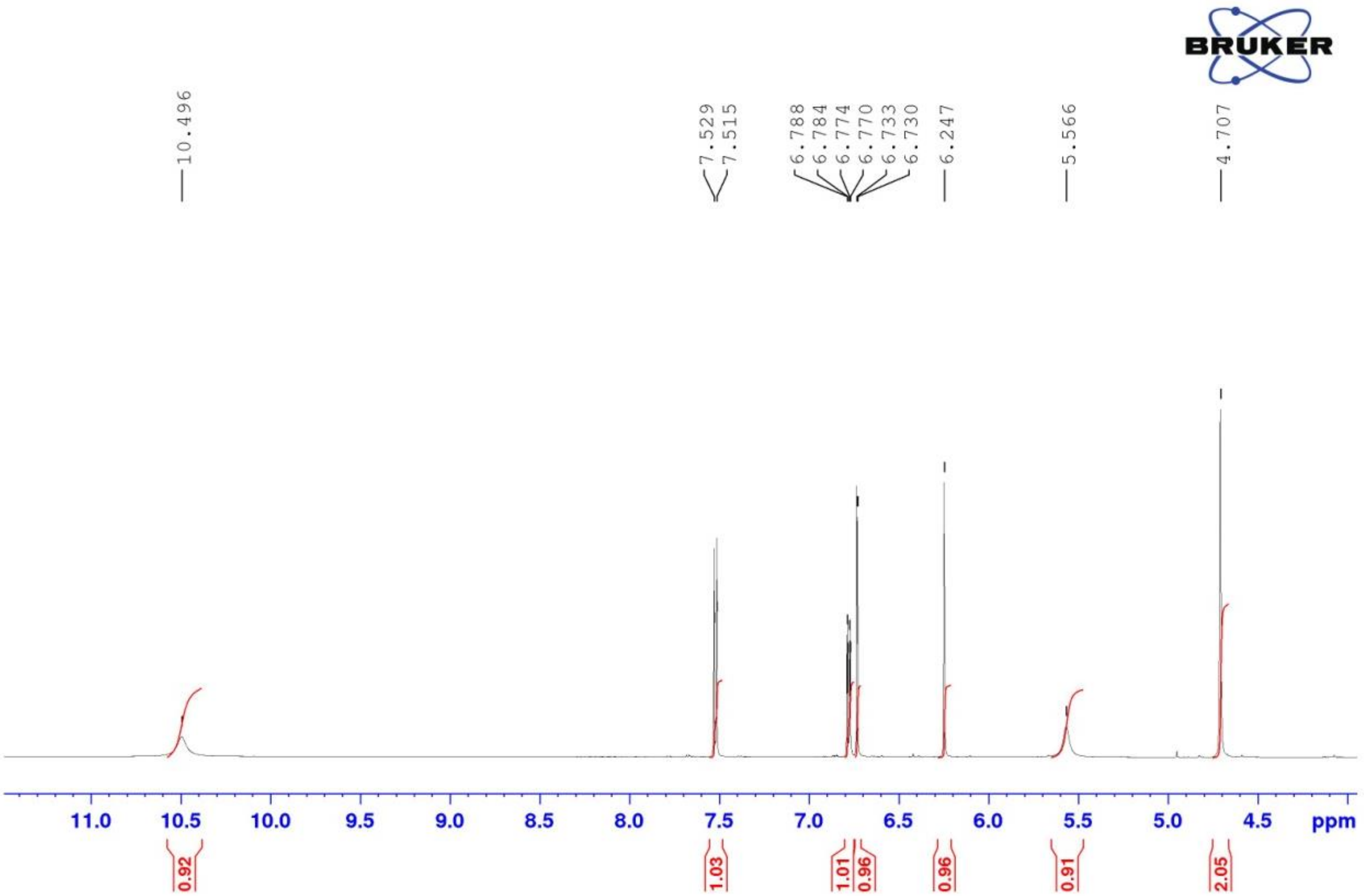

Figure S9. ^{1}H NMR spectrum of **$CmCH_2OH$**

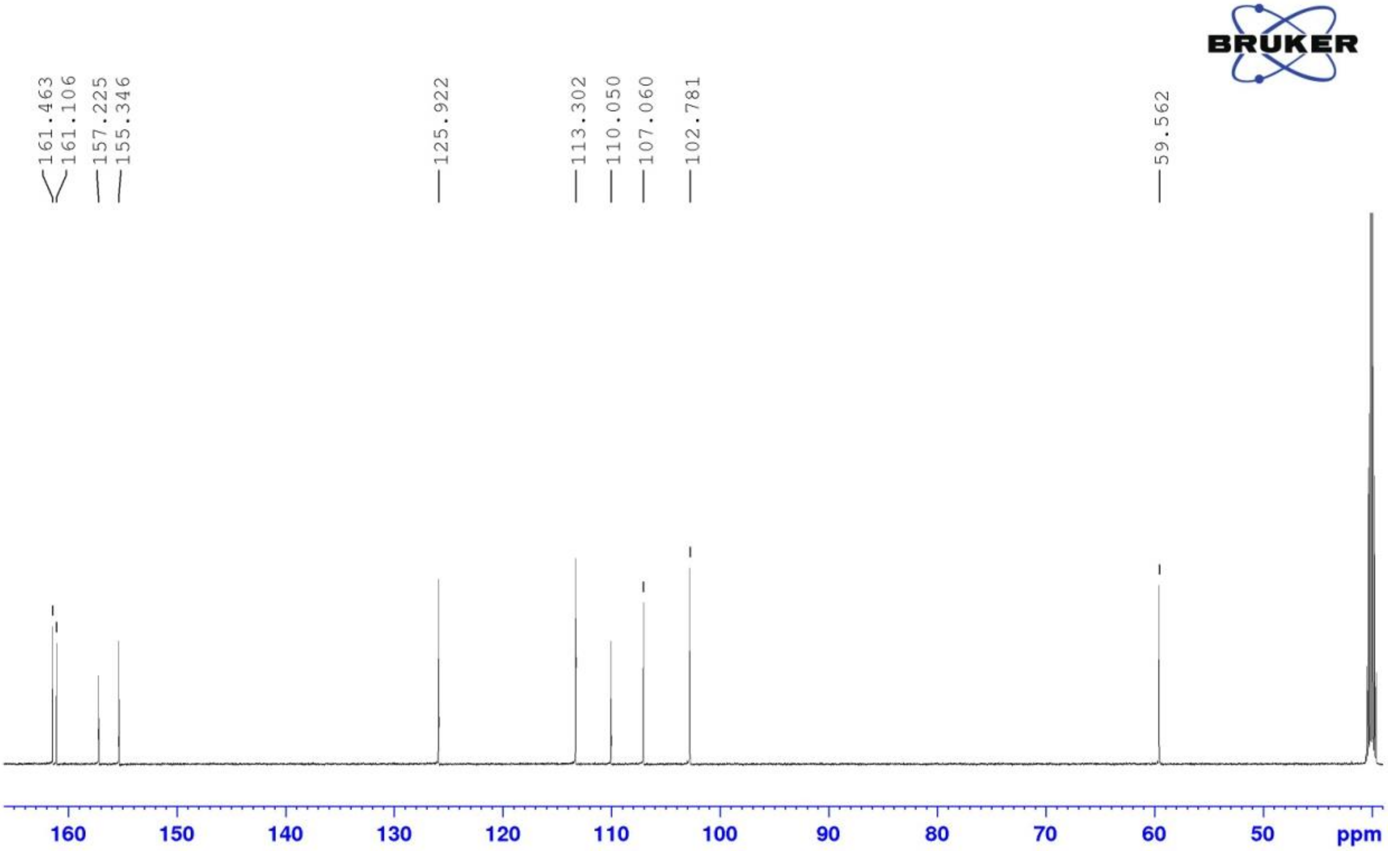

Figure S10. ^{13}C NMR spectrum of **$CmCH_2OH$**

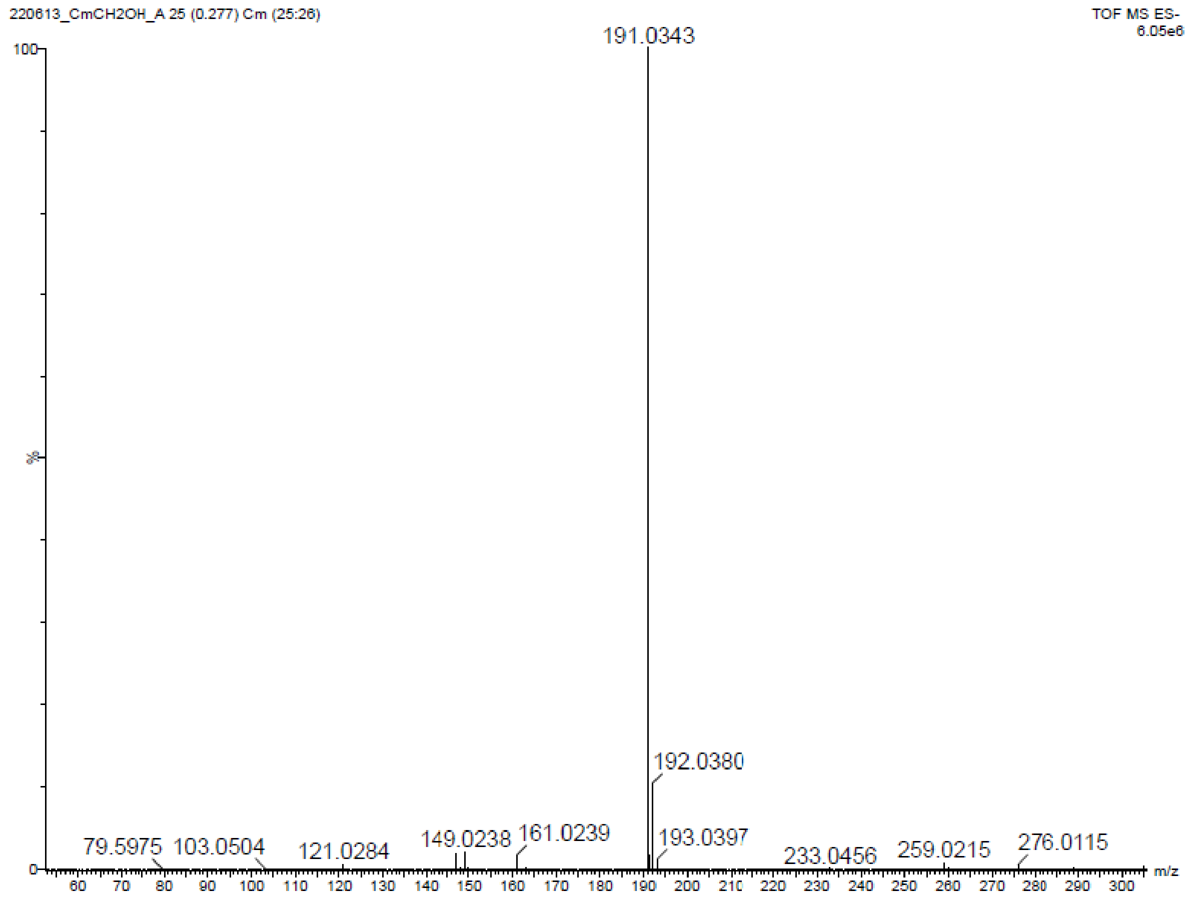

Figure S11. HRMS spectrum of **$CmCH_2OH$**

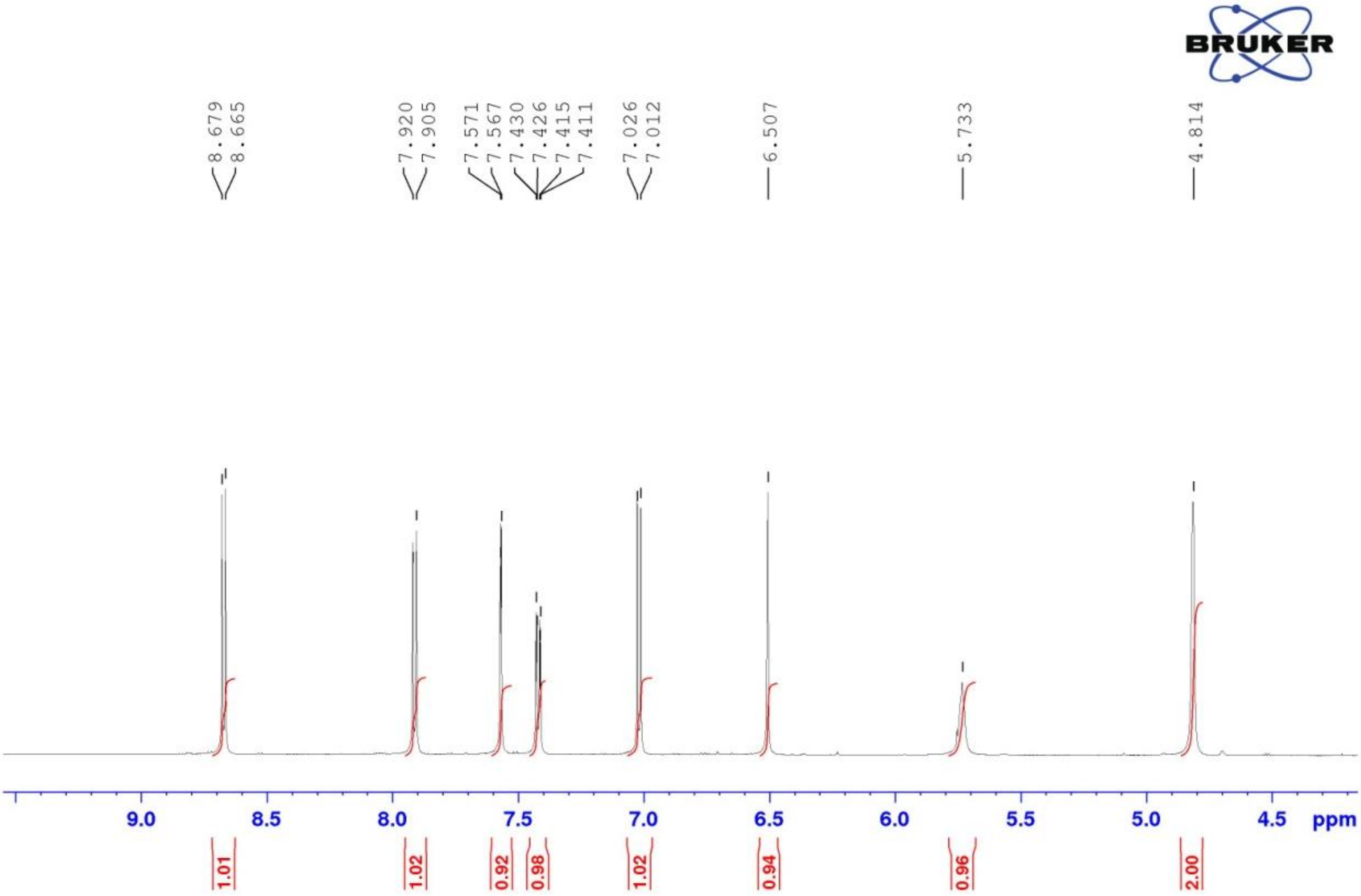

Figure S12. ^{1}H NMR spectrum of **NBD-O-CmCH$_2$OH**

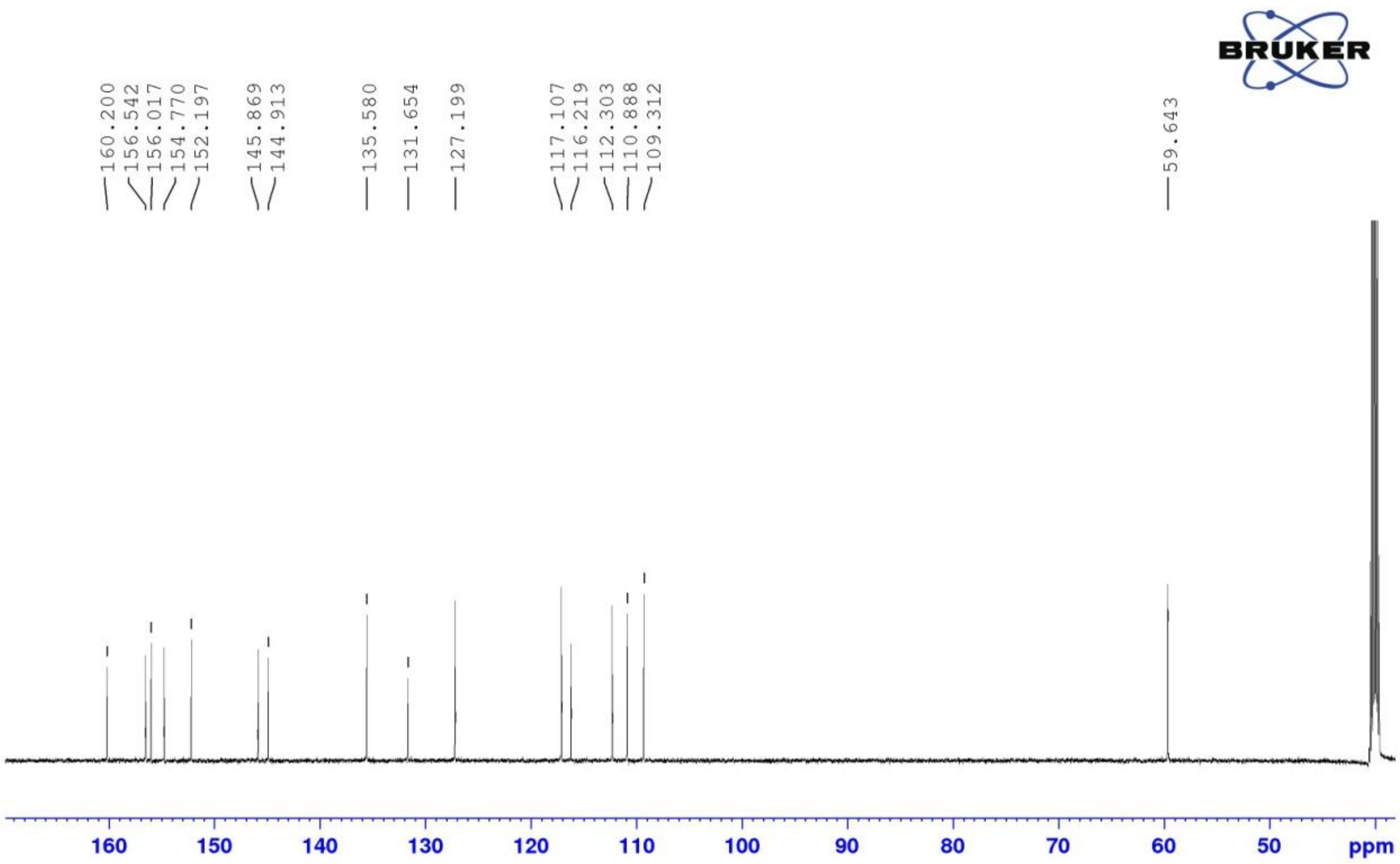

Figure S13. ^{13}C NMR spectrum of **NBD-O-CmCH$_2$OH**

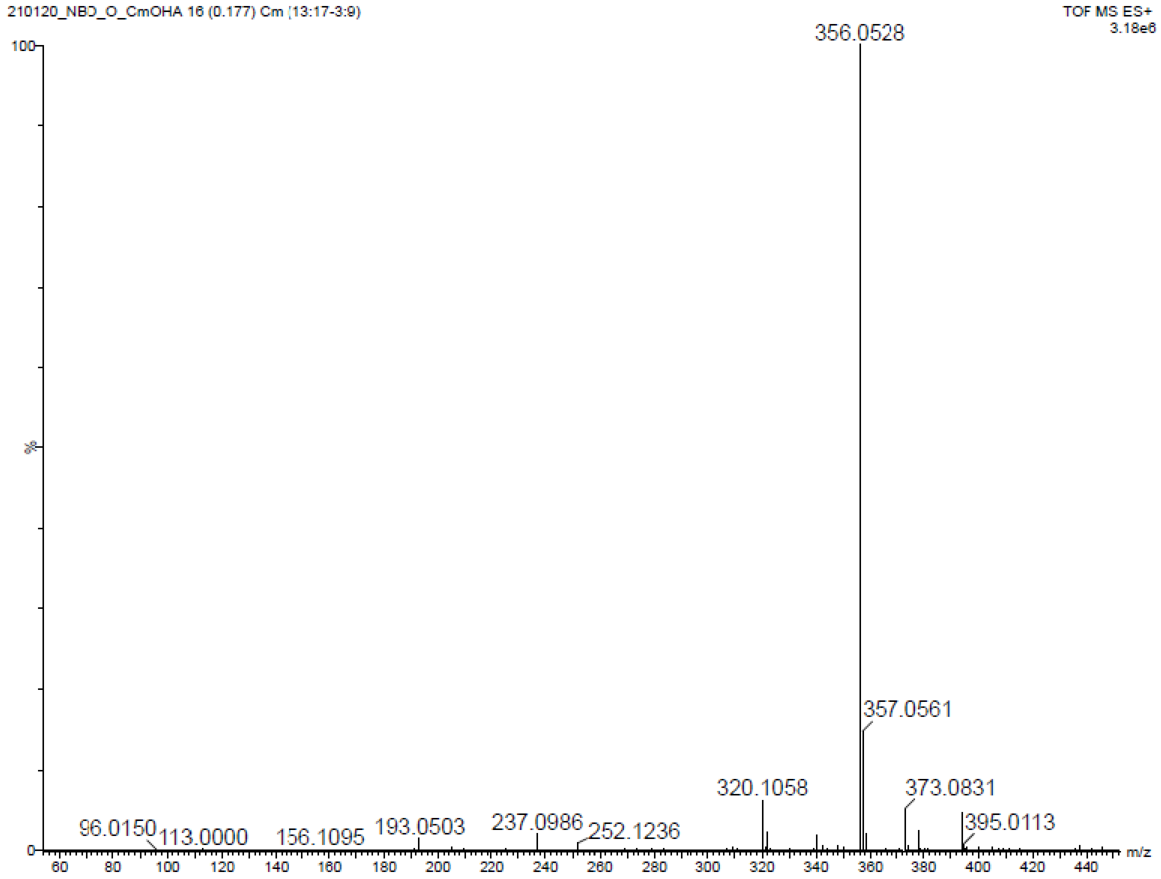

Figure S14. HRMS spectrum of **NBD-O-CmCH$_2$OH**

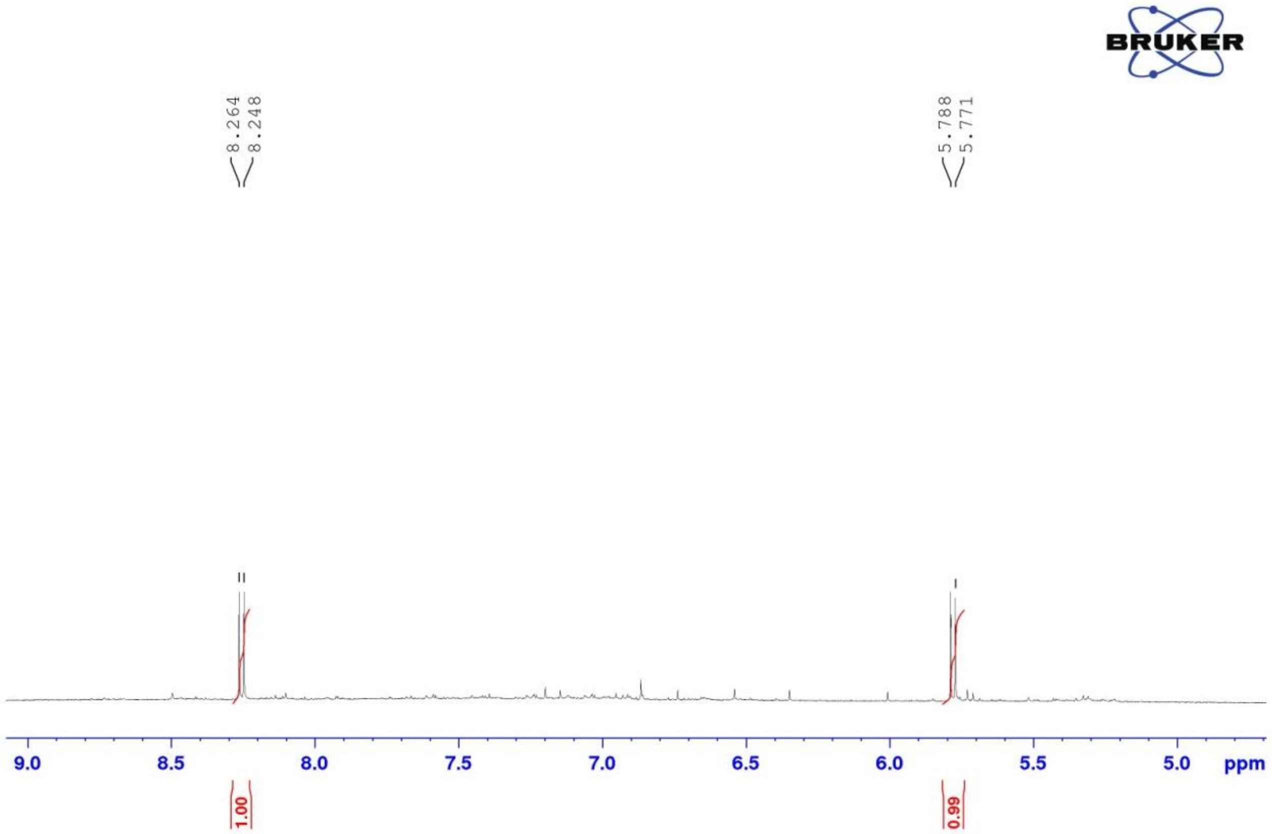

Figure S15. ^{1}H NMR spectrum of **NBD-SH**

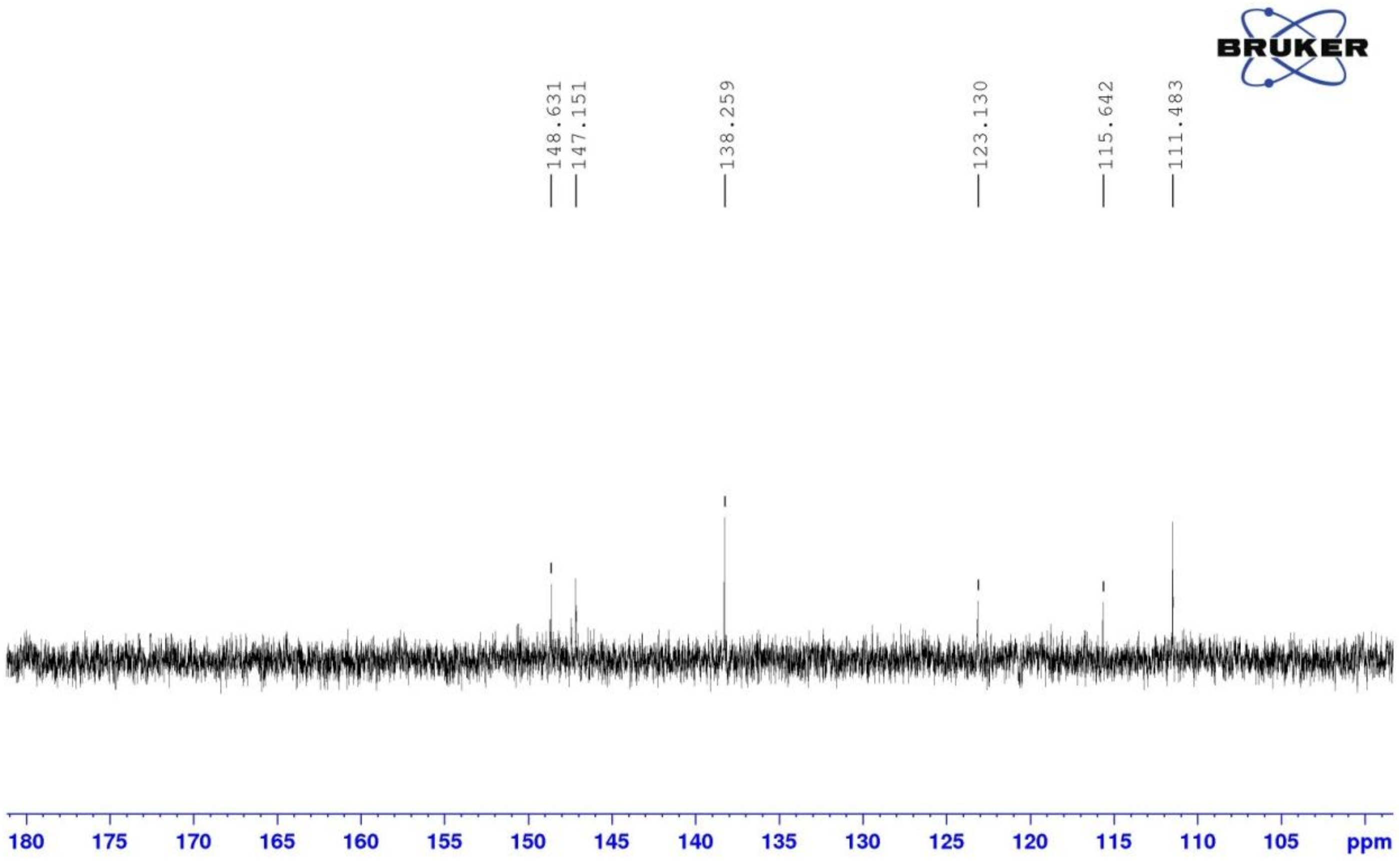

Figure S16. ^{13}C NMR spectrum of **NBD-SH**

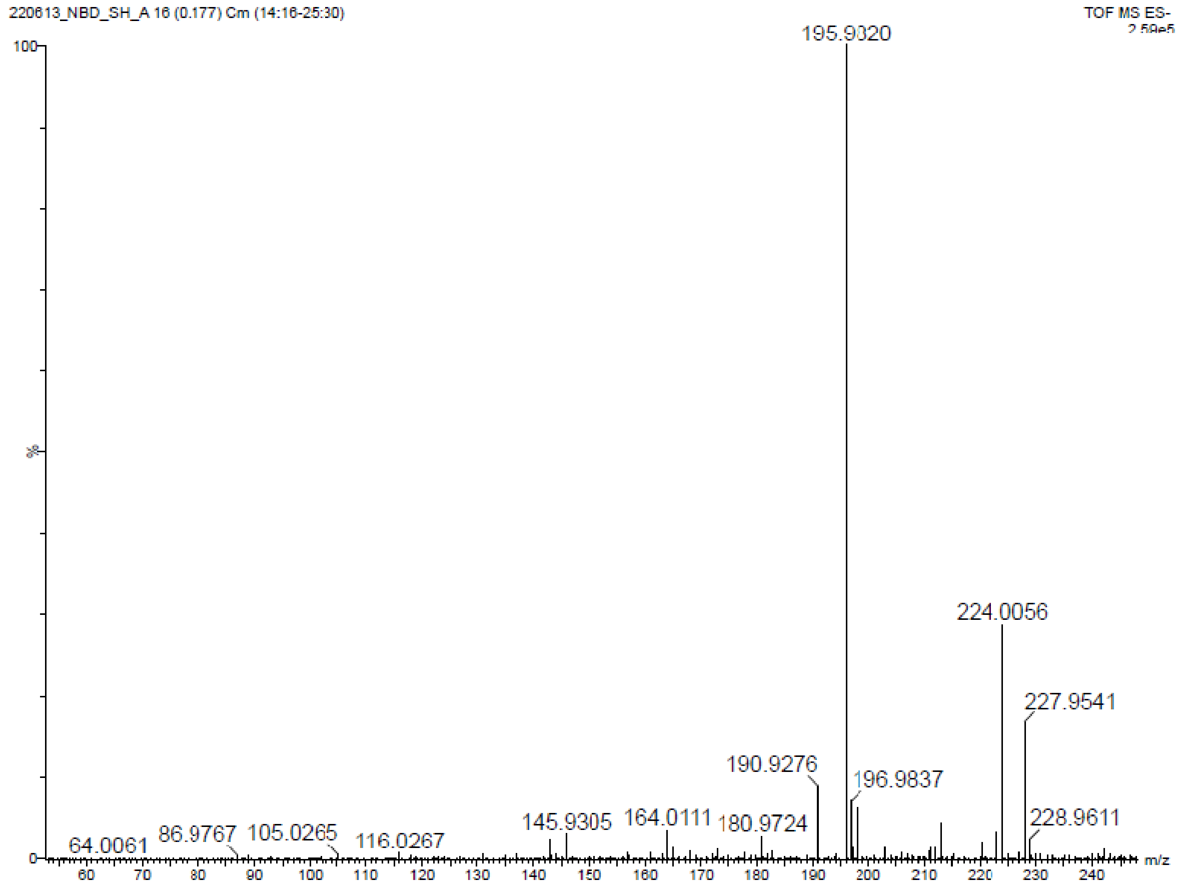

Figure S17. HRMS spectrum of **NBD-SH**

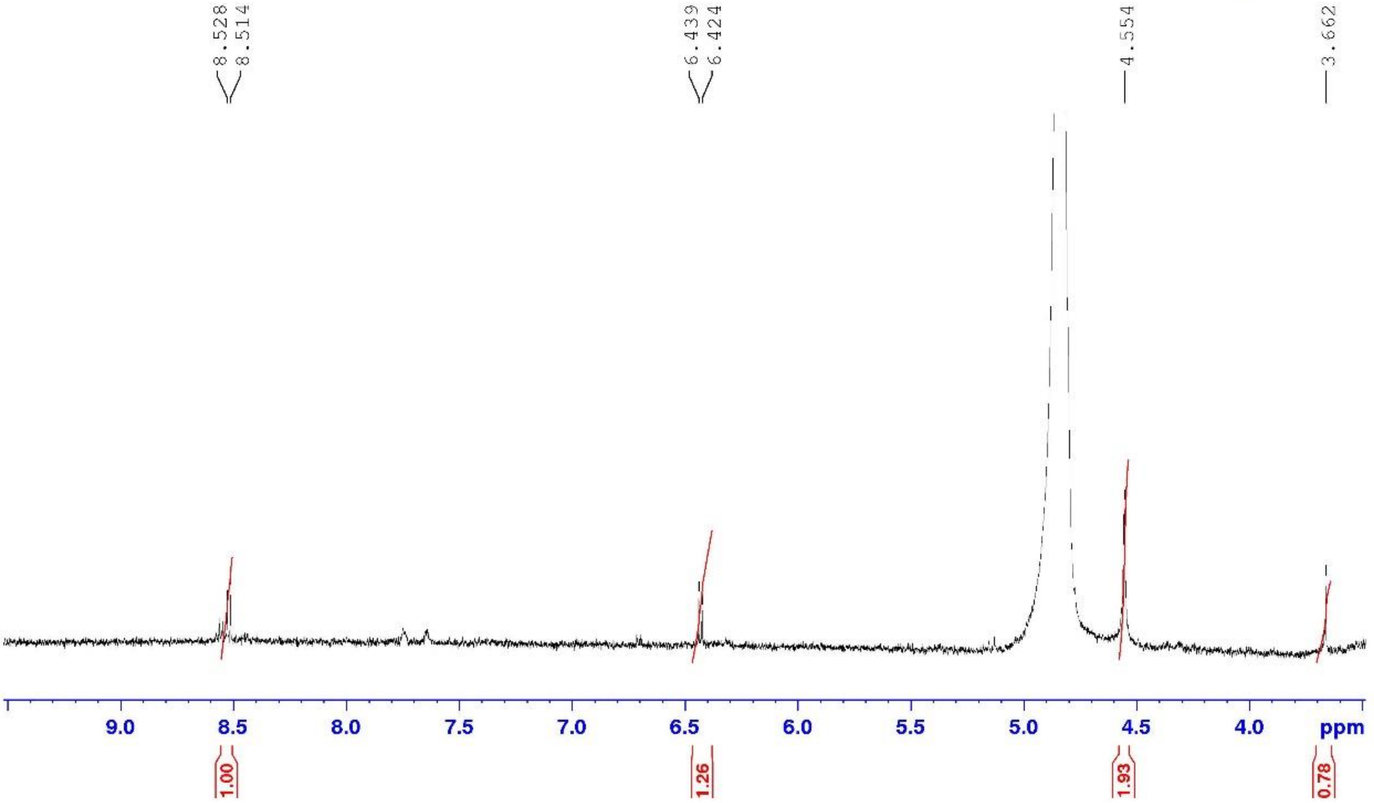

Figure S18. ^{1}H NMR spectrum of **NBD-Cys**

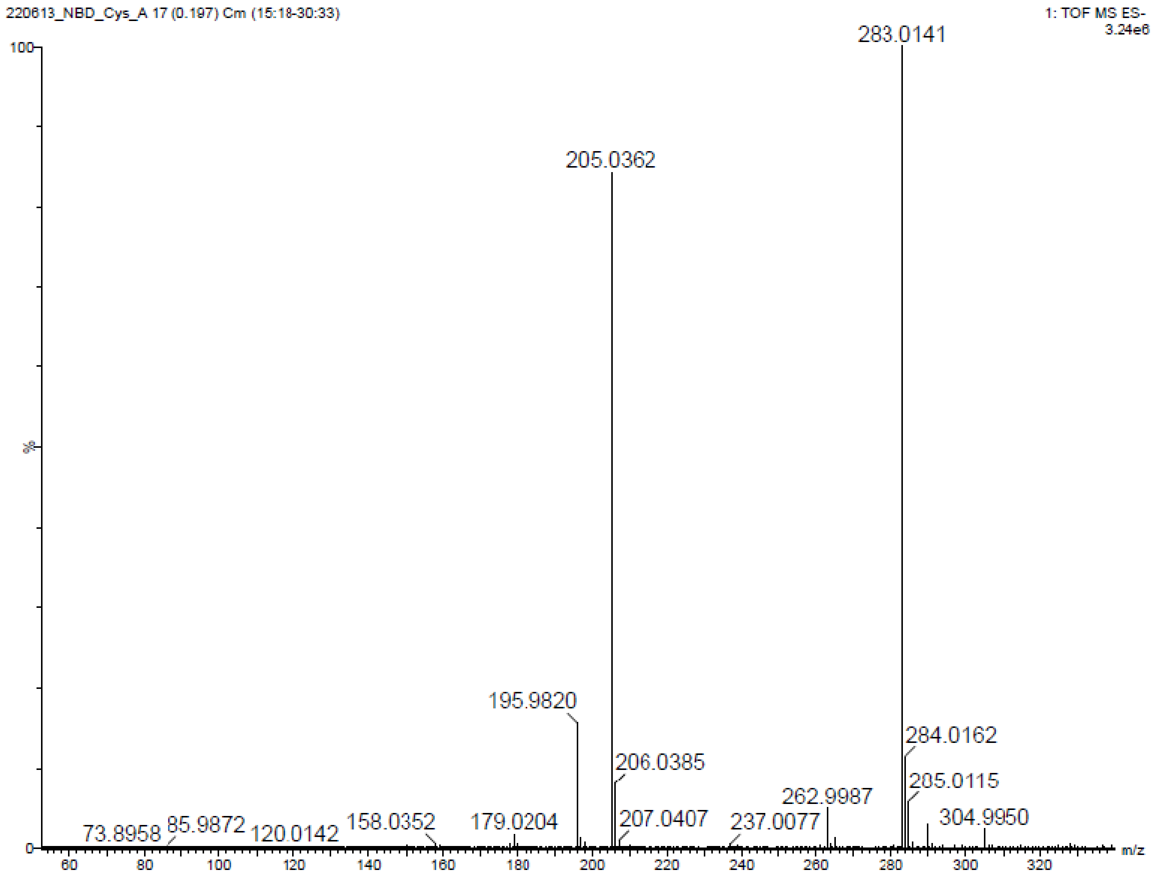

Figure S19. HRMS spectrum of **NBD-Cys**

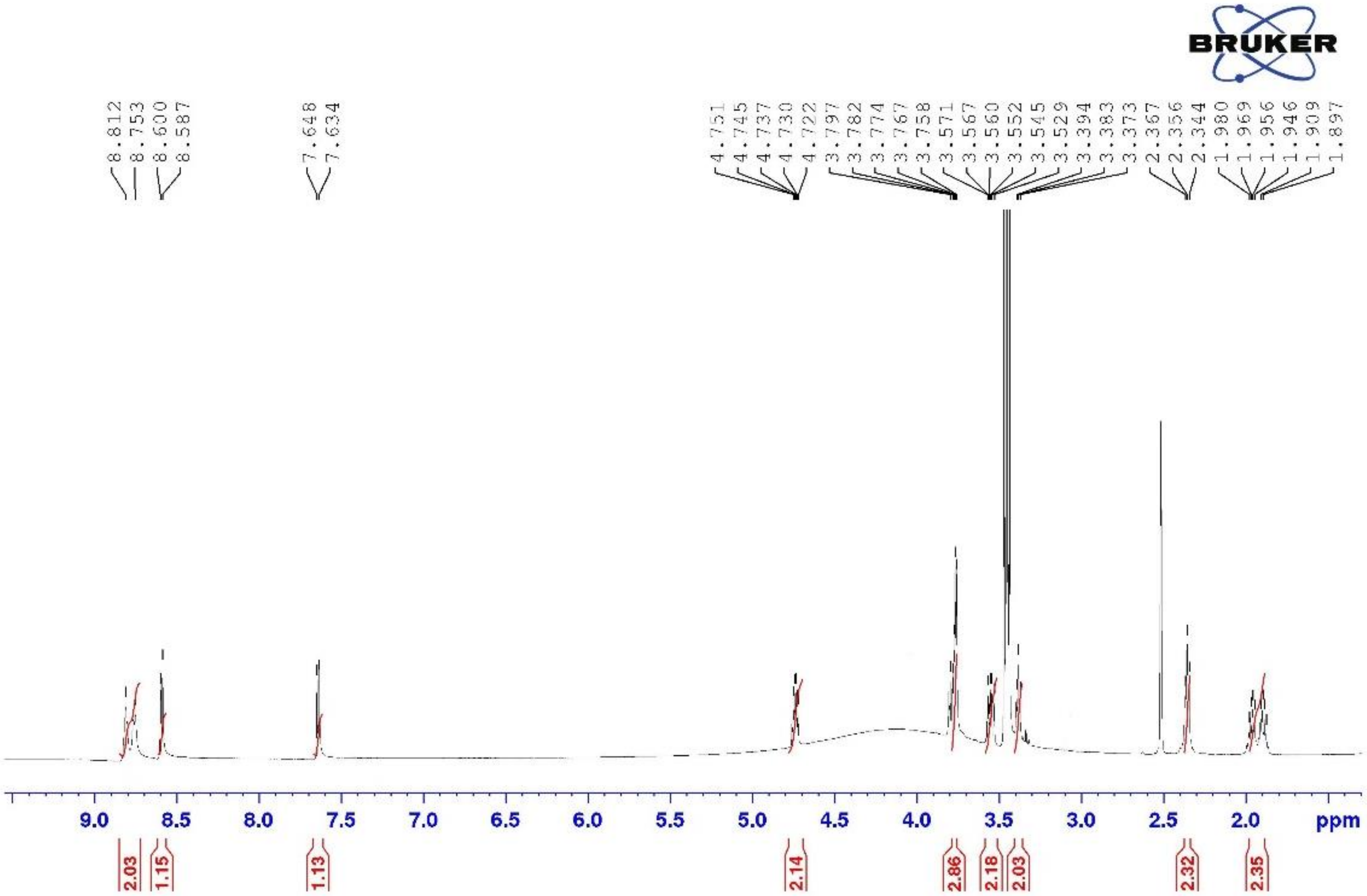

Figure S20. ^{1}H NMR spectrum of **NBD-GSH**

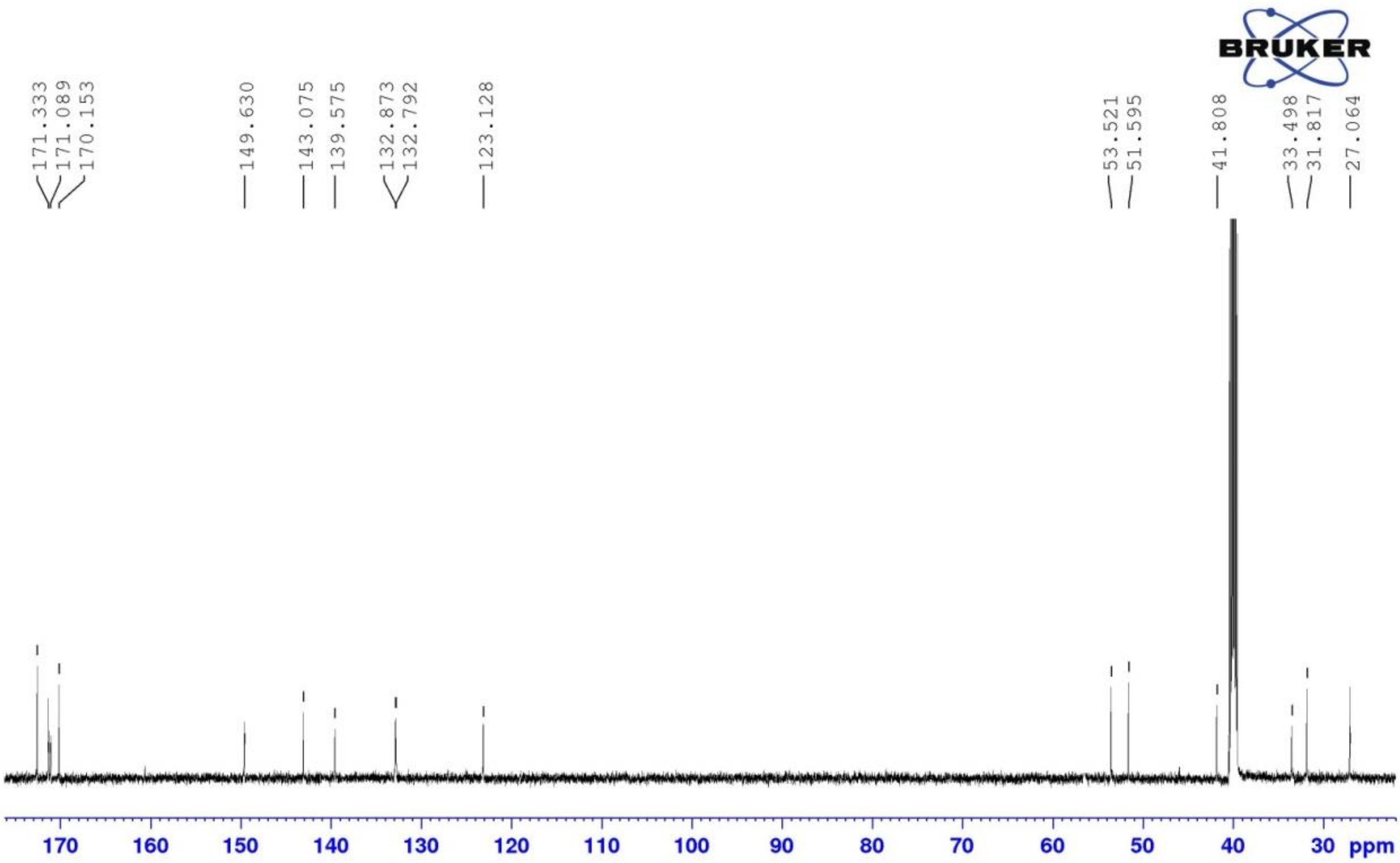

Figure S21. ^{13}C NMR spectrum of **NBD-GSH**

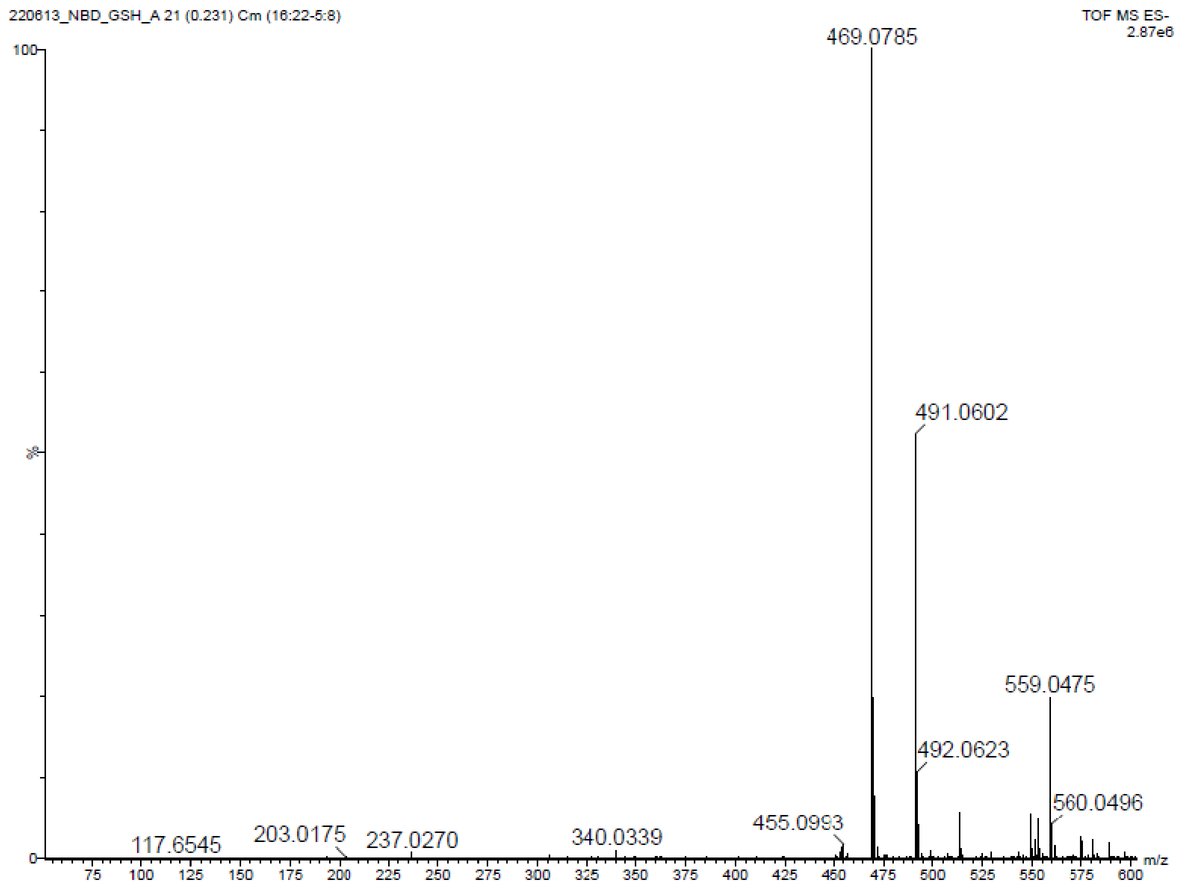

Figure S22. HRMS spectra of **NBD-GSH**

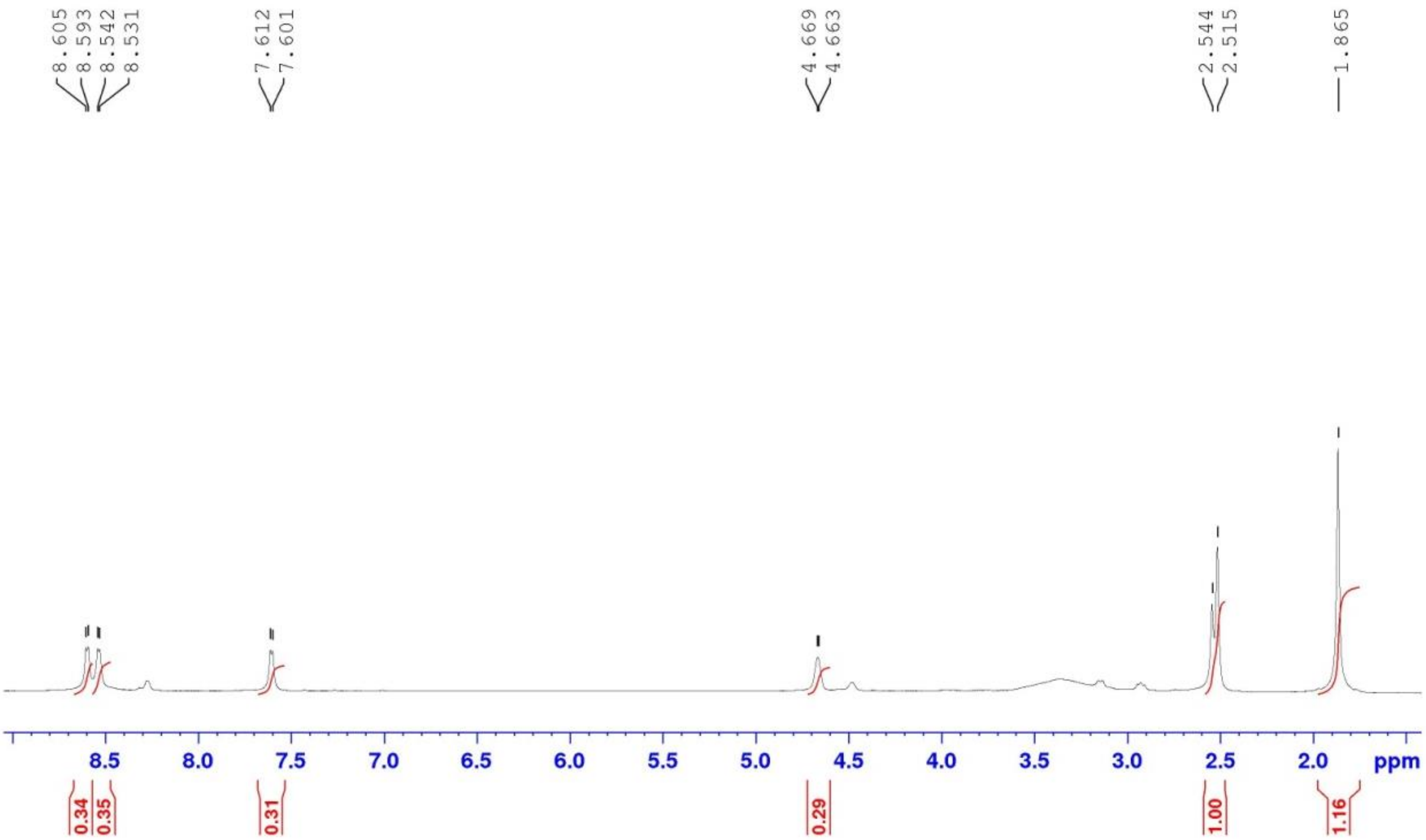

Figure S23. ^{1}HNMR spectrum of **NBD-NAC**

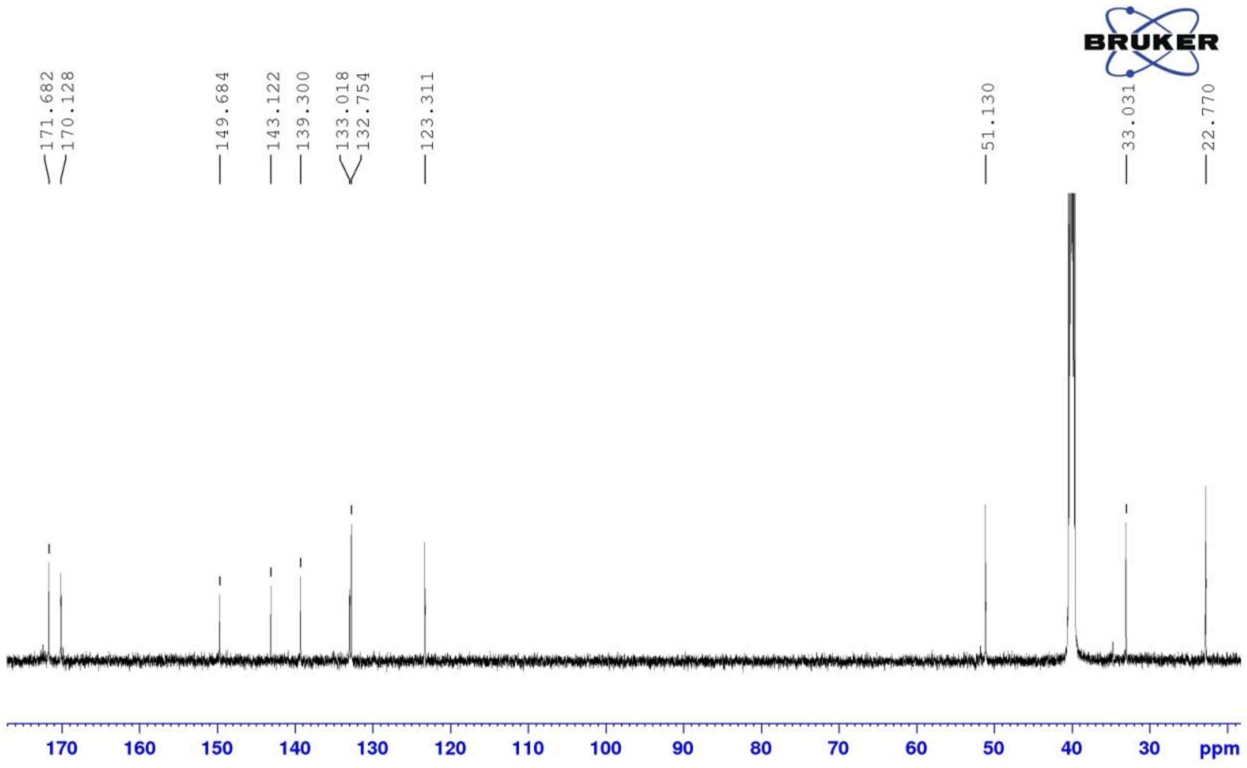

Figure S24. ^{13}C NMR spectrum of **NBD-NAC**

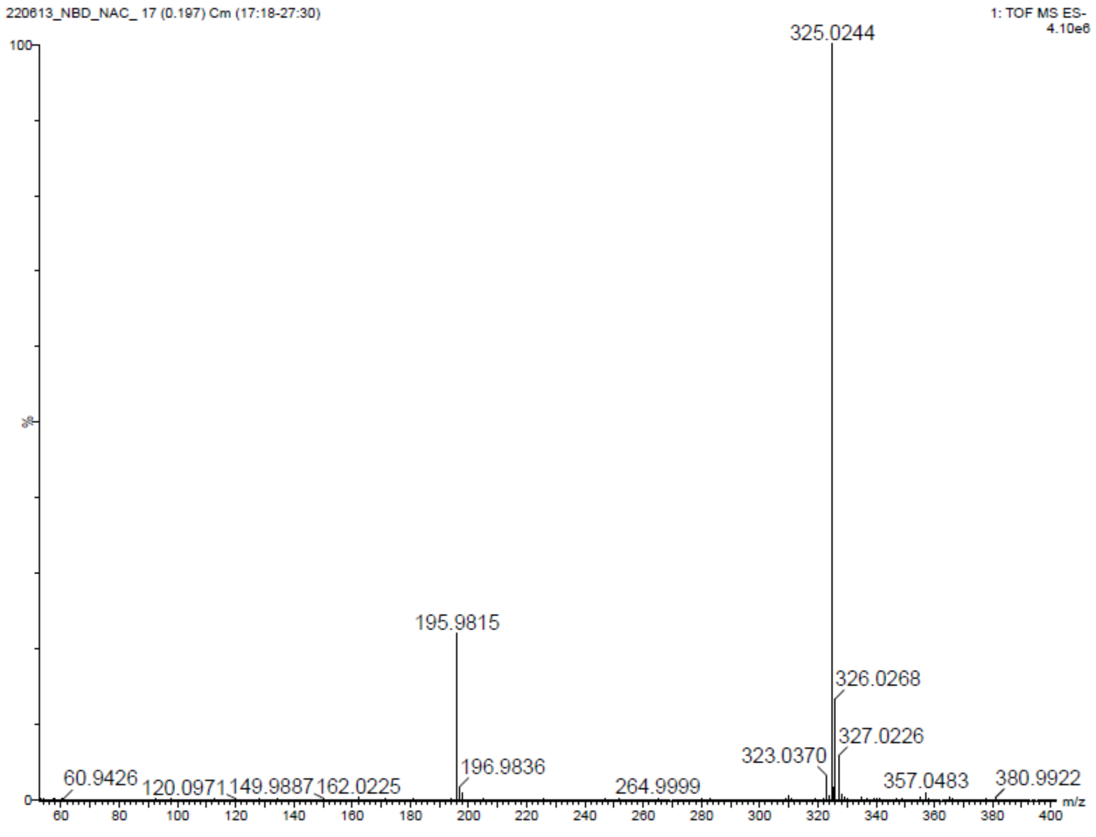

Figure S25. HRMS spectrum of **NBD-NAC**

[S1] Zhang, Y.; Wang, J.; Yue, J.; Chao, J.; Huo, F.; Yin, C. A new strategy for the fluorescence discrimination of Cys/Hcy and GSH/H_2S simultaneously colorimetric detection for H_2S. *Spectrochim. Acta - A: Mol. Biomol.* **2020**, *227*, 117537.

[S2] Chen, W.; Luo, H.; Liu, X.; Foley J. W.; Song, X. Broadly applicable Strategy for the fluorescence based detection and differentiation of glutathione and cysteine/homocysteine: demonstration in vitro and in vivo. *Anal. Chem.* **2016**, *88*, 3638-3646.

[S3] Zhu, H.; Liu, C.; Yuan, R.; Wang, R.; Zhang, H.; Li, Z.; Jia, P.; Zhu, B.; Sheng, W. A simple highly specific fluorescent probe for simultaneous discrimination of cysteine/ homocysteine and glutathione/hydrogen sulfide in living cells and zebrafish using two separated fluorescence channels under single wavelength excitation. *Analyst* **2019,** *144*, 4258

[S4] Liu, T.; Li, S.; Zhang, X.; Wang, J.; Deng, Y.; Sun, X.; Xing, Z.; Wu, R. A facile probe for fluorescence turn-on and simultaneous naked-eyes discrimination of H_2S and biothiols (Cys and GSH) and its application. *J Fluoresc.* **2022**, *32*, 175-188.

[S5] Hao,Y., Zhang, Y.; Zhu, D.; Luo, L.; Chen, L.; Tang, Z.; Zeng, R.; Xu, M.; Chen, S. Dual-emission fluorescent probe for discriminative sensing of biothiols. *Chinese J. Anal. Chem.* **2022**, *50*, 100153.

[S6] Yang, Y.; Xu, Z.; Han, L.; Fan, Y.; Qing, M.; Li, N; Luo, H. A simple fluorescent probe with two different fluorescence signals for rapid sequence distinguishing of Cys/Hcy/GSH and intracellular imaging. *Dyes and Pigments* **2021**, *184*, 108722.

[S7] Xu, S.; Zhou, J.; Dong, X.; Zhao, W.; Zhu, Q. Fluorescent probe for sensitive discrimination of Hcy and Cys/GSH in living cells via dual-emission. *Anal. Chim. Acta.* **2019**, *1074*, 123-130.

[S8] Niu, H.; Duan, Y.; Zhang ,Y.; Hua, X.; Xu, C.; Li, Z.; Ma, J.; Qin, F.; Zhai, Y.; Ye, Y.; Zhao, Y. A bifunctional fluorescent probe based on PET & ICT for simultaneously recognizing Cys and H_2S in living cells. *J. Photochem. Photobiol. B, Biol.* **2022**, *230*, 112441.
